# Impact of the inspiratory oxygen fraction on the cardiac output during jugulo-femoral venoarterial extracorporeal membrane oxygenation in the rat

**DOI:** 10.1186/s12872-022-02613-w

**Published:** 2022-04-15

**Authors:** Fabian Edinger, Emmanuel Schneck, Charlotte Schulte, Goetz Schmidt, Johannes Gehron, Michael Sander, Christian Koch

**Affiliations:** 1 Department of Anesthesiology, Intensive Care Medicine and Pain Therapy, Justus Liebig University of Giessen, Gießen, Germany; 2 Department of Adult and Pediatric Cardiovascular Surgery, Justus Liebig University of Giessen, Gießen, Germany

**Keywords:** ECMO, Cardiogenic shock, Apnea, Ventilation during ECMO, Harlequin phenomenon

## Abstract

**Background:**

Venoarterial extracorporeal membrane oxygenation (V-A ECMO) with femoral access has gained wide acceptance in the treatment of critically ill patients. Since the patient´s cardiac output (CO) can compete with the retrograde aortic ECMO-flow, the aim of this study was to examine the impact of the inspiratory oxygen fraction on the cardiac function during V-A ECMO therapy.

**Methods:**

Eighteen male Lewis rats (350–400 g) received V-A ECMO therapy. The inspiratory oxygen fraction on the ventilator was randomly set to 0.5 (group A), 0.21 (group B), or 0 in order to simulate apnea (group C), respectively. Each group consisted of six animals. Arterial blood pressure, central venous saturation (S_cv_O_2_), CO, stroke volume, left ventricular ejection fraction (LVEF), end diastolic volume, and pressure were measured. Cardiac injury was determined by analyzing the amount of lactate dehydrogenase (LDH).

**Results:**

During anoxic ventilation the systolic, mean and diastolic arterial pressure, CO, stroke volume, LVEF and S_cv_O_2_ were significantly impaired compared to group A and B. The course of LDH values revealed no significant differences between the groups.

**Conclusion:**

Anoxic ventilation during V-A ECMO with femoral cannulation leads to cardiogenic shock in rats. Therefore, awake V-A ECMO patients might be at risk for hypoxia-induced complications.

**Supplementary Information:**

The online version contains supplementary material available at 10.1186/s12872-022-02613-w.

## Background

Extracorporeal membrane oxygenation (ECMO) has been widely accepted for the treatment of pulmonary or cardiac failure in critically ill patients, which has led to increased implementation in intensive care medicine over the last decade [1]. While venoarterial ECMO (V-A ECMO) is used in patients suffering from cardiac failure with and without severe respiratory dysfunction, venovenous ECMO (V-V ECMO) is indicated in patients suffering from severe respiratory failure with preserved cardiac function [2].

In contrast to complete cardiopulmonary bypass (CPB) perfusion with cardioplegic cardiac arrest, during ECMO therapy the heart continues beating [3]. Knowledge of the altered hemodynamic effects of ECMO compared to CPB is crucial for the management of extracorporeal cardiac life support. Particularly, the type and location of arterial and venous cannulation remain essential for adequate therapy. While the venous inflow is mostly realized by the drainage of the right atrium, the arterial return differs according to the patient's condition or for other therapeutic reasons. Due to the favorable risk profile, a percutaneous cannulation is frequently performed by cannulating the femoral artery. However, it has to be recognized that femoral access leads to a retrograde perfusion of the aorta including the coronary and cerebral arteries [2, 4]. Antegrade perfusion is not chosen on a regular basis because it involves open-chest cannulation of aorta, subclavian, or carotid arteries leaving the patient at higher risk of infection, ischemic events, and bleeding [2]. Even though femoral cannulation offers a preferable risk profile, the competition between the retrograde ECMO-perfusion of the aorta and the body´s own cardiac output (CO) leads to the distinct risk of the development of a watershed within the aorta branch [5]. This mechanism is known as the harlequin phenomenon and potentially results in a critical perfusion/oxygenation mismatch of the upper body. Radiologic studies using computed tomography were able to visualize the watershed within the aortic arch [5–7]. Typically, this phenomenon can be observed during pulmonary dysfunction with recovered left ventricular function [6]. This is of particular importance because patients in need of V-A ECMO therapy often also suffer from respiratory complications which is addressed with ultraprotective ventilation strategies [8, 9]. Otherwise, positive pressure ventilation implies the risk of ventilator-induced lung injury (VILI). Particularly, high driving pressures and cyclic re- and decruitment result in severe lung injury. Therefore, the concept of awake ECMO therapy has been developed and offers the opportunity to reduce the incidence of VILI in patients suffering from severe respiratory failure [10–12].

Recent studies showed the beneficial effects of awake V-A ECMO therapy but especially these patients are at an increased risk for the development of ventilator-associated pneumonia [13, 14]. As a result, their pulmonary function might be impaired which is of high clinical importance because arterial femoral cannulation can lead to a harlequin phenomenon in spontaneously breathing ECMO patients. While the respiratory rate is centrally regulated by the arterial partial pressure of carbon dioxide (pCO_2_), both ECMO and mechanical ventilation affect pCO_2_ values. During awake ECMO therapy, a decrease of pCO_2_ might lead to apnea resulting in a severe deoxygenation with consecutive ischemia of the coronary and cerebral tissue caused by the harlequin phenomenon. For this reason, the aim of our study was to examine the impact of the inspiratory oxygen fraction (F_i_O_2_) on cardiac function during V-A ECMO therapy with jugolo-femoral access.

## Methods

### Animals

All procedures involving animals were conducted in compliance with standards for animal care and the ARRIVE guidelines, and were approved by the responsible single local committee for animal care (animal welfare commission of the department of veterinary medicine at the regional council Giessen—GI 20/26 G45/2018; Regierungspraesidium Giessen, Germany).

Twenty-two male Lewis rats (350–400 g) obtained from Janvier Labs (Le Genest St. Isle, France) were housed at 22 °C, 55% relative humidity, and a day/night cycle of 14/10 h, with access to standard chow and water ad libitum. The rats were randomly divided into three groups. The F_i_O_2_ was adjusted at 0.5 (group A, n = 6), 0.21 (group B, n = 6), or 0 in order to simulate apnea (group C; n = 6). Since the measurements of the pressure volume (PV) catheter are influenced by the ventilation, apnea would have affected the results of the PV catheter. Therefore, anoxic ventilation with 100% of nitrogen was performed as a surrogate for simulated apnea.

Four animals had to be euthanized before the end of the study due to perforation of the draining cannula into the chest cavity during the process of cannulation. These animals were excluded from the study.

### Induction and maintenance of anesthesia

After inhalative induction of anesthesia (5% isoflurane (Baxter, Unterschleißheim, Germany) balanced with 100% oxygen) the rats were intubated endotracheally (16 G cannula, B. Braun, Melsungen, Germany) and ventilated volume-controlled (Harvard Inspira, Harvard Apparatus, Cambridge, UK) in a weight-adjusted manner (tidal volume (ml) = mass (kg)^1.01^ × 0.0062; respiratory rate (/minute) = mass (kg)^−0.26^ × 53.5). The animals were placed on an automated heating pad and a rectal temperature probe was inserted. Monitoring included end-tidal carbon dioxide (MicroCapStar, CWE, Ardmore, PA, USA), continuous electrocardiogram, heart rate (HR), CO, stroke volume (SV), left ventricular end diastolic volume (LVEDV) and pressure (LVEDP), left ventricular ejection fraction (LVEF), and arterial blood pressure (systolic, diastolic and mean). After the percutaneous cannulation of the lateral tail vein (24 G cannula, B. Braun, Melsungen, Germany) a continuous balanced crystalloid infusion at a rate of 5 ml/kg/h (Sterofundin B. Braun, Melsungen, Germany) with fentanyl (10 µg/kg/h, Albrecht GmbH, Aulendorf, Germany) was started.

Additionally, midazolam (2 mg/kg/h, Roche, Basel, Switzerland) and pancuronium (0.1 mg/kg/h, Inresa, Freiburg, Germany) were administered for the maintenance of anesthesia.

### Extracorporeal membrane oxygenation

The vascular accesses were placed surgically and consisted of the tail artery cannula for measurement of the arterial blood pressure and intermittent blood gas analysis (24 G, B. Braun, Melsungen, Germany), the right femoral artery cannula for ECMO return (22 G catheter, Terumo, Eschborn, Germany), and the right jugular vein cannula for ECMO drainage (modified multi-orifice 17 G cannula, B. Braun, Melsungen, Germany) [15]. Further, for the continuous measurement of left ventricular pressure and volume, a 2 F PV catheter (SPR-838, Millar, Houston, TX, USA) was placed into the left ventricle through the right carotid artery. Prior to cannulation of the right jugular vein, the animals received a heparin bolus (400 IU/kg, Merckle GmbH, Blaubeuren, Germany) through the lateral tail vein.

As previously described, the ECMO circuit consisted of a venous reservoir (M. Humbs, Valley, Germany), a roller-pump (Verderflex Vantage 3000, Castleford, UK), and a membrane oxygenator (Micro-1, Kewei Rising Medical, Shenzhen, China) [15]. The whole circuit was primed with 250 international units of Heparin (Ratiopharm, Ulm, Germany) and 9 ml hydroxy ethyl starch 6% (Voluven, Fresenius Kabi, Bad Homburg, Germany). At first, the blood flow was initiated at a rate of 45 ml/kg/minute and then continuously increased to 90 ml/kg/minute. Subsequently, the F_i_O_2_ was adjusted to 0.5, 0.21 or 0, accordingly. Sweep gas flow on the membrane was regulated between 20 and 30 ml/minute to adjust the pCO_2_ levels between 35 and 45 mmHg. The oxygen fraction on the ECMO membrane was set to 1.0. Further, the animals received no vasopressor support. Moreover, the volume of blood sampling was replaced with hydroxy ethyl starch 6%.

### Timepoints of hemodynamic measurements

Baseline values were captured before commencing the ECMO after implementation of all cannulas and catheters at baseline (t_0_). While a new timepoint was defined every 7.5 min, arterial blood pressure and invasive hemodynamic parameters were recorded up to 120 min (t_0_–t_16_). Depending on the procedural injury, the animals in groups A and B were observed for two hours, whereas the observation period of group C was one hour due to animal welfare.

### Euthanasia

After the observation period all animals received a bolus of 5 µg fentanyl, 0.8 mg midazolam and 0.04 mg pancuronium. Further, isoflurane was adjusted to 5%. After 5 min, the animals were euthanized by collection of the whole blood through the draining ECMO cannula and removal of the heart and lungs.

### Blood analyses

Blood samples were collected at baseline (t_0_), and every 30 min after commencing the ECMO till t_16_. At each observation point the oxygen partial pressure (pO_2_), pCO_2_, hemoglobin, hematocrit, pH, bicarbonate, base excess (BE), lactate, glucose, sodium, potassium, calcium, and chloride were measured (ABL800, Radiometer, Copenhagen, Denmark). In addition, blood samples were centrifuged at 3000 g for 5 min and the plasma samples were stored at −80 °C for further analysis.

Lactate dehydrogenase (LDH) measurements were performed using a commercial enzyme-linked immune sorbent assay (ELISA) according to the manufacturer's instructions (ab102526, Abcam, Cambridge, UK). The probes were unfrozen only once.

### Statistics

The sample size calculation was performed using R statistical software version 3.6.2 (http://www.r-project.org). A group size of ten animals revealed a measurable difference of a twofold standard deviation regarding the LVEF (alpha and beta error of 0.05 and 0.2, respectively). According to animal welfare regulations, intermittent statistical analyses were performed. Following the regulations of the animal welfare board, the experiments were stopped after six animals of each study group because statistical significance of the results had already been reached. All data are expressed as median with 25th and 75th percentile. Homogeneity of variance was verified with the Levene test. If the test of homogeneity was positive, the ANOVA and post hoc Tukey test were used for comparison of intergroup differences at identical time points, while the Welch and post hoc Games-Howell test were applied during inhomogeneity of variance. Due to the fact that group C ended after t_8_, groups A and B were compared with Wilcoxon–Mann–Whitney test between t_8_ and t_16_. The analysis of variations in LDH levels across different time points within the same group was calculated by the Friedmann test. All statistical analyses were performed using SPSS Version 20 (IBM, Stuttgart, Germany). GraphPad Prism Version 7 was used for data presentation (GraphPad Software, San Diego, CA, USA).

## Results

### Hemodynamic measurements

**Fig. 1:**
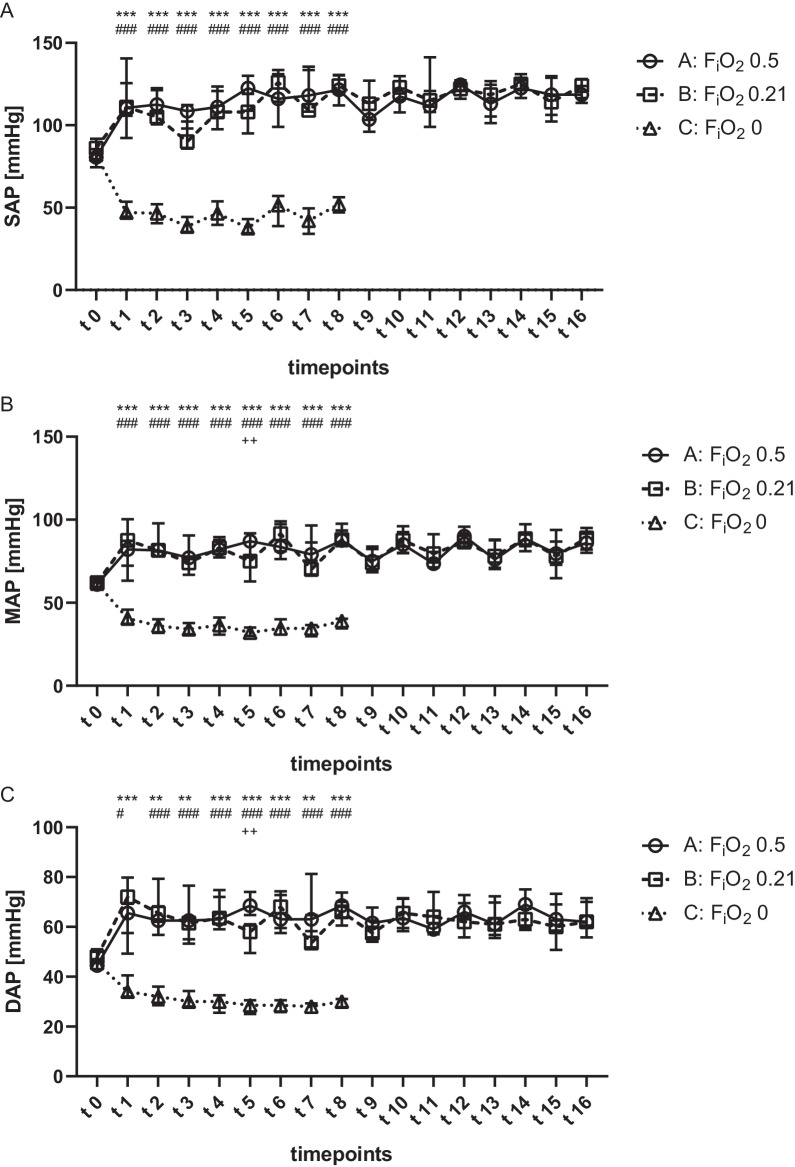
Invasive blood pressure during V-A ECMO. Animals were ventilated with inspiratory oxygen fraction of 0.5 (group A), 0.21 (group B) or 0 (group C). Values were recorded every 7.5 min until the end of the experiment. The systolic (**A**) mean (**B**) and diastolic (**C**) arterial pressure were significantly reduced during anoxic ventilation. Results are presented as median with interquartile range. Asterisks, rhombus and crosses display the degree of statistical significance: A vs. C: **p < 0.01; ***p < 0.001; B vs. C: #*p* < 0.05; ###*p* < 0.001; A vs B: ++*p* < 0.01. Abbreviations: SAP: systolic arterial pressure; MAP: mean arterial pressure; DAP: diastolic arterial pressure; F_i_O_2_: inspiratory oxygen fraction

**Fig. 2:**
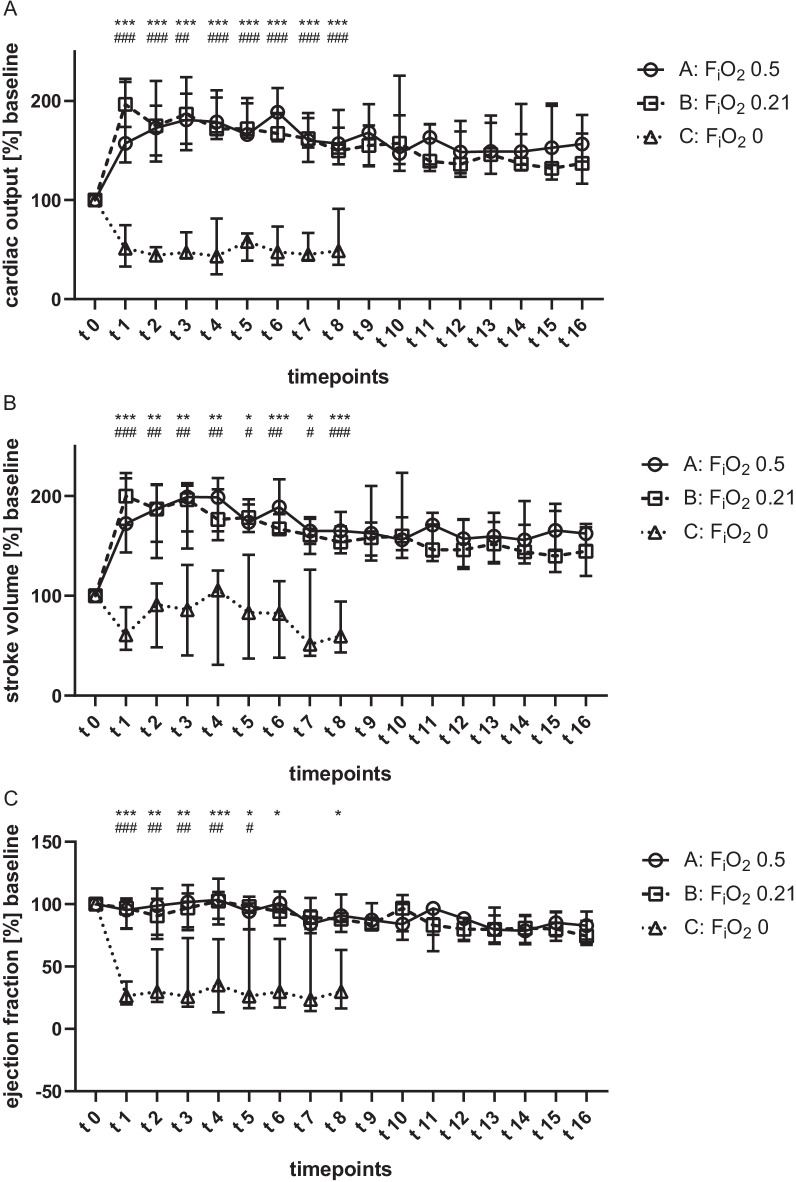
Invasive hemodynamic parameters during V-A ECMO. Animals were ventilated with inspiratory oxygen fraction of 0.5 (group A), 0.21 (group B) or 0 (group C). Values were recorded every 7.5 min until the end of the experiment. The cardiac output (**A**) stroke volume (**B**) and ejection fraction (**C**) were significantly reduced during anoxic ventilation. Results are presented as median with interquartile range. Asterisks, rhombus and crosses display the degree of statistical significance: A vs. C: *p < 0.05; **p < 0.01; ***p < 0.001; B vs. C: ^#^*p* < 0.05; ^##^*p* < 0.01; ^###^*p* < 0.001; A vs B: ^+^*p* < 0.05. Abbreviations: F_i_O_2_: inspiratory oxygen fraction

Compared to groups A (F_i_O_2_ 0.5) and B (F_i_O_2_ 0.21) the systolic, diastolic, and mean arterial pressure were significantly decreased during anoxic ventilation (group C). Contrary, no differences were found between group A and B after commencing the ECMO (Fig. 1). Furthermore, measurements of the HR did not reveal significant differences between groups. While LVEF, SV and CO were significantly impaired during anoxic ventilation (group C), groups A and B did not show alterations of LVEF, SV and CO (Fig. 2). However, LVEDP was significantly reduced in group B at t_5_ and t_15_. Additionally, no differences in LVEDV were found between all groups.

### LDH

**Fig. 3:**
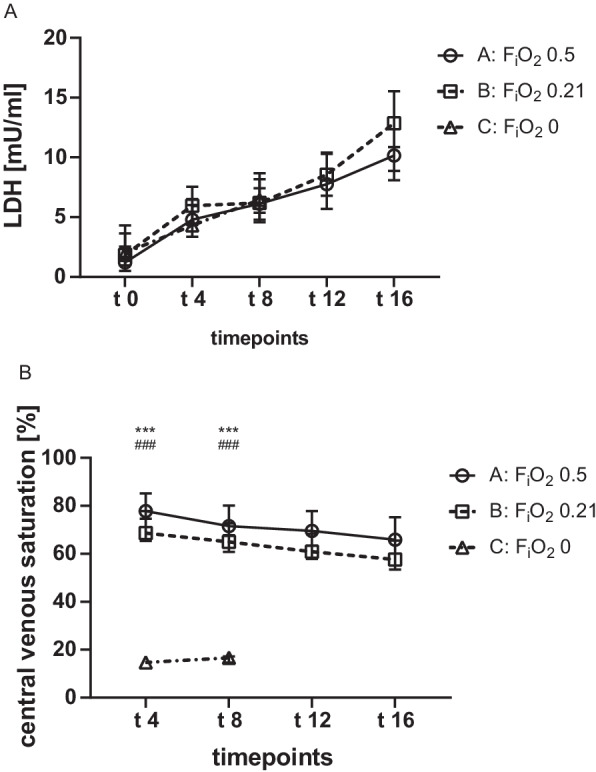
Time course of LDH and Central venous saturation during V-A ECMO. Animals were ventilated with inspiratory oxygen fraction of 0.5 (group A), 0.21 (group B) or 0 (group C). Blood was withdrawn for analysis at baseline and every 30 min after commencing the V-A ECMO. While the LDH (A) was continuously rising in all group, no differences were analyzed between the groups. Anoxic ventilation was associated with a significant decrease of the central venous saturation. Results are presented as median with interquartile range. Asterisks and rhombus display the degree of statistical significance: A vs. C: ***p < 0.001; B vs. C: ^###^*p* < 0.001. Abbreviations: F_i_O_2_: inspiratory oxygen fraction; LDH: lactate dehydrogenase

The measured concentration of LDH was rising continuously within groups B and C during the observation period (A: *p* = 0.199; B: *p* < 0.001; C: *p* = 0.007; Fig. 3A). Further, no significant differences were found between the three groups.

### Blood gas analyses

Significantly lower pO_2_ and elevated pCO_2_ values were measured during pulmonary hypoxia (group C) at t_4_ and t_8_ compared to groups A and B (Table 1). Furthermore, potassium, and calcium were significantly increased in group C at t_4_ and t_8_ (Additional file 1: Table S1), while pH, bicarbonate, and BE were significantly reduced in group C at t_4_ and t_8_ (Table 1). Glucose and lactate were significantly elevated, whereas the central venous saturation (S_cv_O_2_) was significantly reduced in group C during t_4_ and t_8_ (Table 1, Fig. 3B).

**Table 1:** Results of the blood gas analysis

value	Group	t_0_	t_4_	t_8_	t_12_	t_16_
pH	A	7.42 [7.38–7.43]	7.48 [7.46–7.51]	7.48 [7.47–7.50]	7.45 [7.44–7.48]	7.44 [7.42–7.45]
	B	7.40 [7.37–7.42]	7.47 [7.47–7.52]	7.47 [7.46–7.51]	7.45 [7.43–7.48]	7.42 [7.41–7.46]
	C	7.39 [7.37–7.40]	7.19 [7.18–7.24] *** ###	7.15 [7.09–7.18] *** ###		
Bic	A	25.9 [24.4–26.9]	27.0 [26.3–27.8]	26.1 [22.7–27.0]	24.7 [23.7–26.4]	23.8 [22.8–24.2]
(mmol/l)	B	25.2 [24.9–27.2]	27.3 [26.0–27.7]	26.1 [24.9–26.4]	23.8 [22.8–24.2]	23.8 [23.7–24.2]
	C	24.8 [24.0–25.3]	16.3 [14.7–17.5] *** ###	13.8 [12.3–14.8] *** ###		
BE	A	1.7 [−0.1–2.7]	2.9 [2.1–3.8]	2.5 [0.8–3.5]	0.2 [−0.9–2.2]	−0.9 [−1.9–0.3]
(mmol/l)	B	0.8 [0.5–3.1]	3.2 [1.6–3.7]	1.8 [0.5–2.2]	0.6 [−0.2–1.7]	−0.9 [−0.9–0.3]
	C	0.3 [−0.5–1.0]	−10.4 [−12.1–8.6] *** ###	−13.5 [−15.6–12.2] *** ###		
pO_2_	A	169 [151–198]	431 [405–468]	413 [357–431]	417 [359–454]	415 [349–425]
(mmHg)	B	156 [114–206]	399 [353–451]	387 [336–439]	367 [334–402]	349 [299–387]
	C	179 [147–208]	160 [115–213] *** ###	171 [126–239] *** ###		
pCO_2_	A	42 [39–45]	36 [33–36]	34 [32–36]	35 [33–36]	34 [32–36]
(mmHg)	B	42 [40–48]	34 [33–37]	34 [32–35]	35 [33–36]	36 [32–38]
	C	42 [39–44]	43 [42–46] *** ###	42 [42–43] *** ###		
Hb	A	13.7 [13.3–14.0]	7.9 [7.7–8.3]	7.8 [7.4–8.0]	7.6 [7.3–7.8]	7.3 [6.6–7.8]
(g/dl)	B	13.7 [13.5–13.9]	8.5 [7.9–8.6]	8.0 [7.3–8.1]	7.8 [7.3–8.1]	7.6 [7.4–7.8]
	C	13.2 [12.9–13.6]	7.8 [7.6–8.1]	8.0 [7.6–8.2]		
Glu	A	175 [165–180]	139 [133–141]	121 [119–128]	113 [103–119]	107 [101–114]
(mg/dl)	B	179 [170–187]	142 [133–152]	124 [111–140]	124 [110–138]	118 [114–119] + +
	C	184 [166–194]	351 [311–363] *** ###	301 [274–310] *** ###		
Lac	A	1.9 [1.8–2.2]	1.7 [1.5–1.9]	1.2 [1.0–1.4]	1.5 [1.2–1.6]	1.4 [1.4–1.5]
(mmol/l)	B	1.8 [1.6–1.9]	1.7 [1.5–1.9]	1.3 [1.0–1.5]	1.6 [1.3–2.1]	1.7 [1.6–2.0] +
	C	2.1 [1.9–2.3]	9.4 [9.0–9.9] *** ###	11.5 [10.8–13.2] *** ###		

Animals were ventilated with inspiratory oxygen fraction of 0.5 (group A), 0.21 (group B) or 0 (group C). Blood was withdrawn for analysis at baseline and every 30 min after commencing the V-A ECMO. Animals of group C presented a lactate induced acidosis, as well as reduced pO_2_ and increased pCO_2_ levels. Data are shown as median (with 25th and 75th percentile). Asterisks, rhombus and crosses display the degree of statistical significance: A vs. C: *p < 0.05; ***p < 0.001; B vs. C: ^#^*p* < 0.05; ^##^*p* < 0.01; ^###^*p* < 0.001; A vs B: ^+^*p* < 0.05 ^++^*p* < 0.01

*Bic* bicarbonate, *BE* base excess, *pO*_*2*_ arterial partial pressure of oxygen, *pCO*_*2*_ arterial partial pressure of carbone dioxide, *Hb* hemoglobin, *Glu* glucose, *Lac* lactate

## Discussion

This study aimed to investigate the impact of pulmonary hypoxia during anoxic ventilation on the LVEF during V-A ECMO with jugulo-femoral cannulation. Anoxic ventilation (group C) resulted in severe hemodynamic alterations which was defined by a significant reduction of systolic, diastolic, and mean arterial pressure. Furthermore, a significant impairment of cardiac function determined by a reduction of LVEF and CO was observed during pulmonary hypoxia, which was mainly induced by a decrease of the SV but not the HR indicating cardiac failure.

Even though these results seem unsurprisingly, they are the first to describe systemically the relevance of apnea on the cardiac function during ECMO-therapy with femoral cannulation because commonly rat models of CPB are performed under asphyctic cardiac or deep hypothermic circulatory arrest [16–19]. Furthermore, the majority of studies did not measure LVEF or CO. Even though Ali et al. used a PV catheter in a rat model of V-A ECMO with femoral cannulation, their methodology was significantly different. Due to different study aims, the animals primarily underwent 15 min of hypoxic cardiac arrest before eventually commencing the ECMO and ventilation [20]. Fujii et al. recently published another rodent model of V-A ECMO; however, since arterial cannulation was performed through the carotid artery, the circulatory impact of the V-A ECMO differed from our model with femoral cannulation [21]. Even though Kato et al. demonstrated that the coronary blood flow is inversely proportional to the ECMO flow in a V-A ECMO model with carotid access, this is not necessarily applicable to femoral access with retrograde aortic perfusion [22].

Cardiogenic shock during anoxic ventilation was reflected by the severely impaired S_cv_O_2_ and increased lactate level. Next, loss of BE, decreased bicarbonate, and impaired pH demonstrated that the acid–base metabolism was not able to compensate for the lactate induced acidosis. These results are in accordance with the findings of Engels et al. who investigated rats under circulatory arrest. Despite the deep hypothermic circulatory arrest, bicarbonate levels of 13.8 mmol/l and a BE of -12.5 were measured during rewarming [19]. In addition, the elevated ionized calcium during pulmonary hypoxia is most likely caused by the acidosis. Since metabolic acidosis leads to insulin resistance, this might also explain the elevated glucose levels during pulmonary hypoxia [23].

Lower blood pO_2_ and elevated pCO_2_ are also explainable with the anoxic ventilation within study group C. However, Koning et al. were able to adjust the pO_2_ between 150 and 250 mmHg within a rat model of CPB with femoral cannulation and apnea before the beginning of CPB [17]. These studies included blood flow rates of 150–200 ml/kg/minute which were higher than rates of 90 ml/kg/minute during our experiments. It must be highlighted that our approach focused on compensation of a anoxic ventilation during V-A ECMO with femoral cannulation and continuous CO by a F_i_O_2_ of 1.0 during ECMO therapy.

Due to the fact that the ventilator driving pressure during ECMO therapy is associated with increased in-hospital mortality, ultraprotective lung ventilation under ECMO-supported oxygenation is widely adopted across ECMO centers [9, 24, 25]. Since V-V ECMO therapy displays the commonly used treatment option during severe respiratory failure, most studies concentrated on V-V ECMO rather than on V-A ECMO therapy. However, contrary to V-A ECMO with femoral cannulation, V-V ECMO leads to sufficient lung perfusion with oxygenated blood and, therefore, neither cardiac nor cerebral ischemia is to be expected. Since mechanical ventilation is known to induce lung injury by barotrauma and volutrauma, current approaches focus on awake ECMO without mechanical ventilation [11, 12]. For this reason, awake V-A ECMO therapy with cardiocirculatory indication offers a promising therapeutic option but also leaves the patients at risk for apnea [26–29]. Following this, our results indicate that these patients might be vulnerable to cardiac ischemia during apnea. Caused by the harlequin phenomenon these adverse effects were independent of the 100% oxygen insufflation of the ECMO. Following this, the oxygen content of the coronary arteries seems to depend highly on the ventilatory F_i_O_2_. Therefore, the impaired cardiac function during anoxic ventilation appears to be caused by the low oxygen saturation of the coronary blood. However, to the knowledge of the authors, it is technically not possible to measure the coronary blood flow in rats. Further, it offers the risk of exsanguination due to additional aortic cannulation and probe placement. Last, the measurement of both—coronary blood flow and blood saturation—would have required an open chest cavity, which affects the measurements of the pressure volume catheter. We decided against increasing the ECMO flow because an enhanced ECMO flow might raise the already reduced mean arterial pressure and, thus, aggravate the shock signs by raising the left ventricular afterload. This is shown by Konig et al., even though they used no PV catheter in their model. In the description of their experiments, the blood pressure of the tail artery showed an almost flat line during CPB indicating low CO [17]. Contrarily, we were always able to detect a pulsatile arterial wave in the tail artery during V-A ECMO therapy. Furthermore, the LVEDV did not differ between the experimental groups, implying that the ECMO-induced watershed has not increased the afterload or moved towards the heart. Moreover, experiments with an in-vitro mock circulation loop demonstrated that an increased ECMO flow was unable to shift the watershed towards the aortic arch [30]. In summary, this study underlines the importance to avoid apnea during awake V-A ECMO with femoral cannulation in order to lower the risk for pulmonary and coronary hypoxia. For the same reason, it is crucial to monitor the respiratory rate and to adjust the sweep gas flow on the V-A ECMO restrictive to avoid hypocapnia.

Our experiments still have some limitations. First, positive intrathoracic pressures induced by the mechanical ventilation are lacking during awake V-A ECMO with spontaneous breathing. However, it is very difficult to perform good quality left ventricular PV measurements during spontaneous breathing. Furthermore, passive oxygenation with ambient air can occur during apnea. Since no passive oxygenation is possible during ventilation with a F_i_O_2_ of 0, our approach is different from apnea. Compared to apnea without ventilation, we opted for continuous ventilation without oxygen to maintain the ventilation-induced positive intrathoracic pressure. While the reduced intrathoracic pressure during spontaneous breathing could enhance the perfusion of the lungs and thus affect the left ventricular PV measurements, our experimental groups would not have been comparable. Therefore, we also opted for ventilation during V-A ECMO with neuromuscular blocking to avoid spontaneous breathing. Moreover, due to the limited diameter of the venous cannula, only blood flows of 90 ml/kg/minute were achieved. Third, this study was not able to proof cardiac ischemia in terms of increasing LDH blood levels only during anoxic ventilation. Lacking of data on the harlequin effect during V-A ECMO therapy in the rat, we designed this study as a pilot study and performed the sample size calculation on the primary endpoint “LVEF”. To reduce unnecessary harm to the animals, we stopped the inclusion of further rodents after the interim analysis according to the regulations of the local department of animal welfare (see statistical analysis). As a result, only a limited number of animals were available for the analyses of secondary endpoints such as the measurement of LDH. For this reason, the study is not able to clarify if a higher number of animals would have revealed significant alterations of the plasma LDH. Furthermore, LDH was rising continuously in all study groups indicating the increasing ECMO-induced cell damage. Clements et al. analyzed the time course of LDH and Troponin I levels after isoproterenol-induced myocardial injury in the rat and revealed serum peak levels of LDH after four and troponin I after three hours, respectively [31]. Following this, the LDH was not significantly different in our experiments due to the short distance between myocardial damage and measurement of LDH. Therefore, we opted against measurement of troponin I. However, histological examination of cardiomyocytes may be useful to clarify this issue in further studies. Moreover, potassium was elevated during pulmonary hypoxia, which might be a surrogate of myocardial damage. Nevertheless, the lactate-induced acidosis could also be causative. Since the function of the lungs could be impaired during V-A ECMO therapy, histological examinations of the lungs could bring more insights into this topic in following experiments. Further, the impact of F_i_O_2_ on the brain during V-A ECMO therapy could be investigated by histological investigations in the future. Last, results from an animal model cannot be directly transferred to humans.

## Conclusion

In summary, the impact of F_i_O_2_ on cardiac function during V-A ECMO with femoral cannulation was investigated. Pulmonary hypoxia led to hypotension and severe cardiac dysfunction determined by a lactate-induced acidosis and severely impaired S_cv_O_2_. For this reason, apnea induced pulmonary hypoxia might endanger patients for cardiac ischemia during V-A ECMO with femoral cannulation.

## Supplementary Information

**Additional file 1**. **Supplementary Table S1:** Results of the blood gas analysis.

## Data Availability

The datasets used and analyzed during the current study are available from the corresponding author upon reasonable request.

## Abbreviations

BE: Base excess
CO: Cardiac output
CPB: Cardiopulmonary bypass
ECMO: Extracorporeal membrane oxygenation
ELISA: Enzyme-linked immune sorbent assay
F_i_O_2_: Inspiratory oxygen fraction
HR: Heartrate
LDH: Lactate dehydrogenase
LVEDP: Left ventricular end diastolic pressure
LVEDV: Left ventricular end diastolic volume
LVEF: Left ventricular ejection fraction
pCO_2_: Carbon-dioxide partial pressure
pO_2_: Oxygen partial pressure
PV: Pressure volume
S_cv_O_2_: Central venous saturation
SV: Stroke volume
V-A: Venoarterial
VILI: Ventilator-induced lung injury

## References

[1] Hill JD, O'Brien TG, Murray JJ, Dontigny L, Bramson ML, Osborn JJ, Gerbode F. Prolonged extracorporeal oxygenation for acute post-traumatic respiratory failure (shock-lung syndrome). Use of the Bramson membrane lung. N Engl J Med. 1972;286:629–34. doi:10.1056/NEJM197203232861204. 10.1056/NEJM197203232861204 5060491

[2] Makdisi G, Wang I-W (2015). Extra corporeal membrane oxygenation (ECMO) review of a lifesaving technology. J Thorac Dis.

[3] Gall A, Follin A, Cholley B, Mantz J, Aissaoui N, Pirracchio R (2018). Veno-arterial-ECMO in the intensive care unit: from technical aspects to clinical practice. Anaesthesia Crit Care Pain Med.

[4] Choi MS, Sung K, Cho YH (2019). Clinical pearls of venoarterial extracorporeal membrane oxygenation for cardiogenic shock. Korean Circ J.

[5] Hoeper MM, Tudorache I, Kühn C, Marsch G, Hartung D, Wiesner O (2014). Extracorporeal membrane oxygenation watershed. Circulation.

[6] Napp LC, Brehm M, Kühn C, Schäfer A, Bauersachs J (2015). Heart against veno-arterial ECMO: competition visualized. Int J Cardiol.

[7] Cheong C, Xie A, Chew H, Shah M, Shehab S, MacDonald P (2017). Investigation of watershed areas during femoro-femoral Venoarterial Extracorporeal Membrane Oxygenation (VA-ECMO) using a mock loop circuit. J Heart Lung Transplant.

[8] Roumy A, Liaudet L, Rusca M, Marcucci C, Kirsch M (2020). Pulmonary complications associated with veno-arterial extra-corporeal membrane oxygenation: a comprehensive review. Crit Care (London, England).

[9] Schmidt M, Pham T, Arcadipane A, Agerstrand C, Ohshimo S, Pellegrino V, et al. Mechanical ventilation management during extracorporeal membrane oxygenation for acute respiratory distress syndrome. An international multicenter prospective cohort. Am J Respir Crit Care Med. 2019;200:1002–12. doi:10.1164/rccm.201806-1094OC. 10.1164/rccm.201806-1094OC 31144997

[10] Ghadiali S, Huang Y (2011). Role of airway recruitment and derecruitment in lung injury. Crit Rev Biomed Eng.

[11] Gattinoni L, Marini JJ, Collino F, Maiolo G, Rapetti F, Tonetti T (2017). The future of mechanical ventilation: lessons from the present and the past. Crit Care.

[12] Langer T, Santini A, Bottino N, Crotti S, Batchinsky AI, Pesenti A, Gattinoni L (2016). "Awake" extracorporeal membrane oxygenation (ECMO): pathophysiology, technical considerations, and clinical pioneering. Critical care (London, England).

[13] Bouglé A, Bombled C, Margetis D, Lebreton G, Vidal C, Coroir M (2018). Ventilator-associated pneumonia in patients assisted by veno-arterial extracorporeal membrane oxygenation support: Epidemiology and risk factors of treatment failure. PLoS ONE.

[14] Montero S, Huang F, Rivas-Lasarte M, Chommeloux J, Demondion P, Bréchot N (2021). Awake venoarterial extracorporeal membrane oxygenation for refractory cardiogenic shock. Eur Heart J Acute Cardiovasc Care.

[15] Edinger F, Schneck E, Schulte C, Gehron J, Mueller S, Sander M, Koch C (2020). Comparison of the effect of membrane sizes and fibre arrangements of two membrane oxygenators on the inflammatory response, oxygenation and decarboxylation in a rat model of extracorporeal membrane oxygenation. BMC Cardiovasc Disord.

[16] Samarska IV, Henning RH, Buikema H, Bouma HR, Houwertjes MC, Mungroop H (2013). Troubleshooting the rat model of cardiopulmonary bypass: effects of avoiding blood transfusion on long-term survival, inflammation and organ damage. J Pharmacol Toxicol Methods.

[17] Koning NJ, de Lange F, Vonk ABA, Ahmed Y, van den Brom CE, Bogaards S (2016). Impaired microcirculatory perfusion in a rat model of cardiopulmonary bypass: the role of hemodilution. Am J Physiol Heart Circ Physiol.

[18] Magnet IAM, Ettl F, Schober A, Warenits A-M, Grassmann D, Wagner M (2017). Extracorporeal life support increases survival after prolonged ventricular fibrillation cardiac arrest in the rat. Shock.

[19] Engels M, Bilgic E, Pinto A, Vasquez E, Wollschläger L, Steinbrenner H (2014). A cardiopulmonary bypass with deep hypothermic circulatory arrest rat model for the investigation of the systemic inflammation response and induced organ damage. J Inflamm.

[20] Ali AA, Downey P, Singh G, Qi W, George I, Takayama H (2014). Rat model of veno-arterial extracorporeal membrane oxygenation. J Transl Med.

[21] Fujii Y, Tatsumi E, Nakamura F, Oite T (2020). PaO2 greater than 300 mmHg promotes an inflammatory response during extracorporeal circulation in a rat extracorporeal membrane oxygenation model. J Thorac Dis.

[22] Kato J, Seo T, Ando H, Takagi H, Ito T (1996). Coronary arterial perfusion during venoarterial extracorporeal membrane oxygenation. J Thorac Cardiovasc Surg.

[23] Baldini N, Avnet S (2019). The effects of systemic and local acidosis on insulin resistance and signaling. IJMS.

[24] Gattinoni L, Tonetti T, Quintel M (2017). How best to set the ventilator on extracorporeal membrane lung oxygenation. Curr Opin Crit Care.

[25] Serpa Neto A, Schmidt M, Azevedo LCP, Bein T, Brochard L, Beutel G (2016). Associations between ventilator settings during extracorporeal membrane oxygenation for refractory hypoxemia and outcome in patients with acute respiratory distress syndrome: a pooled individual patient data analysis : Mechanical ventilation during ECMO. Intensive Care Med.

[26] Schmidt F, Jack T, Sasse M, Kaussen T, Bertram H, Horke A (2015). "Awake veno-arterial extracorporeal membrane oxygenation" in pediatric cardiogenic shock: a single-center experience. Pediatr Cardiol.

[27] Sommer W, Marsch G, Kaufeld T, Röntgen P, Beutel G, Tongers J (2015). Cardiac awake extracorporeal life support-bridge to decision?. Artif Organs.

[28] Wang H, Jia M, Mao B, Hou X (2017). Atelectasis after airway extubation during veno-arterial extracorporeal membrane oxygenation support. Perfusion.

[29] Deng L, Xia Q, Chi C, Hu G (2020). Awake veno-arterial extracorporeal membrane oxygenation in patients with perioperative period acute heart failure in cardiac surgery. J Thorac Dis.

[30] Gehron J, Schuster M, Rindler F, Bongert M, Böning A, Krombach G (2020). Watershed phenomena during extracorporeal life support and their clinical impact: a systematic in vitro investigation. ESC heart failure.

[31] Clements P, Brady S, York M, Berridge B, Mikaelian I, Nicklaus R (2010). Time course characterization of serum cardiac troponins, heart fatty acid-binding protein, and morphologic findings with isoproterenol-induced myocardial injury in the rat. Toxicol Pathol.

