# Supplementary material for: Impact of the inspiratory oxygen fraction on the cardiac output during jugulo-femoral venoarterial extracorporeal membrane oxygenation in the rat
Source: BMC Cardiovasc Disord. 2022 Apr 15;22:174. doi: 10.1186/s12872-022-02613-w (PMC9013166; doi:10.1186/s12872-022-02613-w)
Supplement: Supplementary file 1 — Additional file 1. Supplementary Table S1: Results of the blood gas analysis. [file 12872_2022_2613_MOESM1_ESM.docx]

**Supplementary Table S1**: Results of the blood gas analysis

| **value** | **group** | **t_0_** | **t_4_** | **t_8_** | **t_12_** | **t_16_** |
| --- | --- | --- | --- | --- | --- | --- |
| S_cv_O_2_ | A |  | 78 [75-85] | 72 [68-80] | 70 [63-78] | 66 [61-75] |
| (%) | B |  | 69 [65-78] | 65 [61-73] | 61 [58-68] | 58 [53-66] |
|  | C |  | 15 [12-16] *** ### | 17 [15-17] *** ### |  |  |
| Hct | A | 42 [41-43] | 25 [24-26] | 24 [23-25] | 23 [23-24] | 23 [20-24] |
| (%) | B | 42 [41-43] | 26 [25-27] | 25 [23-25] | 24 [23-25] | 24 [23-24] |
|  | C | 41 [40-42] | 24 [24-25] | 25 [24-26] |  |  |
| Na^+^ | A | 142 [141-150] | 141 [139-142] | 141 [140-142] | 142 [141-143] | 142 [141-143] |
| (mmol/l) | B | 141 [140-142] | 140 [140-141] | 140 [140-142] | 142 [141-142] | 141 [140-142] |
|  | C | 143 [141-143] | 141 [140-141] | 143 [142-144] * # |  |  |
| K^+^ | A | 3.9 [3.8-4.2] | 3.9 [3.9-4.0] | 3.9 [3.8-4.0] | 3.9 [3.8-4.1] | 3.9 [3.9-4.2] |
| (mmol/l) | B | 4.0 [3.7-4.1] | 3.9 [3.8-4.0] | 3.9 [3.8-4.0] | 4.0 [3.9-4.0] | 4.1 [3.9-4.3] |
|  | C | 4.1 [3.8-4.2] | 4.6 [4.5-4.8] *** ### | 4.9 [4.7-5.3] *** ### |  |  |
| Ca^++^ | A | 1.44 [1.39-1.55] | 1.43 [1.43-1.44] | 1.43 [1.41-1.45] | 1.47 [1.45-1.47] | 1.48 [1.46-1.50] |
| (mmol/l) | B | 1.41 [1.39-1.42] | 1.43 [1.43-144] | 1.44 [1.41-1.45] | 1.47 [1.44-1.47] | 1.46 [1.45-1.48] |
|  | C | 1.41 [1.39-1.44] | 1.47 [1.46-1.50] * ## | 1.52 [1.49-1.53] *** ### |  |  |
| Cl^-^ | A | 105 [104-107] | 108 [107-108] | 107 [106-109] | 108 [107-109] | 107 [107-109] |
| (mmol/l) | B | 106 [105-107] | 108 [108-1109] | 108 [107-108] | 109 [108-109] | 109 [107-109] |
|  | C | 106 [105-108] | 109 [108-110] | 110 [110-111] * # |  |  |

Data are shown as median (with 25^th^ and 75^th^ percentile). Asterisks, rhombus and crosses display the degree of statistical significance: A vs. C: *: *p* < 0.05; ***: *p* < 0.001; B vs. C: #: *p* < 0.05; ## *p* < 0.01; ###: *p* < 0.001; A vs B: +: *p* < 0.05 ++: *p* < 0.01. Abbreviations: S_cv_O_2_: central venous saturation; Hct: hematocrit; Na^+^: sodium; K^+^: potassium; Ca^++^: calcium; Cl^-^: chloride
